# Medical Image Interpolation Using Recurrent Type-2 Fuzzy Neural Network

**DOI:** 10.3389/fninf.2021.667375

**Published:** 2021-09-01

**Authors:** Jafar Tavoosi, Chunwei Zhang, Ardashir Mohammadzadeh, Saleh Mobayen, Amir H. Mosavi

**Affiliations:** ^1^Department of Electrical Engineering, Ilam University, Ilam, Iran; ^2^Structural Vibration Control Group, Qingdao University of Technology, Qingdao, China; ^3^Department of Electrical Engineering, University of Bonab, Bonab, Iran; ^4^Future Technology Research Center, National Yunlin University of Science and Technology, Douliou, Taiwan; ^5^Faculty of Civil Engineering, Technische Universität Dresden, Dresden, Germany; ^6^Institute of Software Design and Development, Obuda University, Budapest, Hungary

**Keywords:** recurrent neural network, type-2 fuzzy system, image interpolation, 2D to 3D, brain MRI, artificial intelligence, machine learning

## Abstract

Image interpolation is an essential process for image processing and computer graphics in wide applications to medical imaging. For image interpolation used in medical diagnosis, the two-dimensional (2D) to three-dimensional (3D) transformation can significantly reduce human error, leading to better decisions. This research proposes the type-2 fuzzy neural networks method which is a hybrid of the fuzzy logic and neural networks as well as recurrent type-2 fuzzy neural networks (RT2FNNs) for advancing a novel 2D to 3D strategy. The ability of the proposed methods in the approximation of the function for image interpolation is investigated. The results report that both proposed methods are reliable for medical diagnosis. However, the RT2FNN model outperforms the type-2 fuzzy neural networks model. The average squares error for the recurrent network and the typical network reported 0.016 and 0.025, respectively. On the other hand, the number of fuzzy rules for the recurrent network and the typical network reported 16 and 22, respectively.

## Introduction

In medical imaging, a cross-sectional sequence of high-resolution organs or tissues is obtained using CT, MRI, or other methods (Leng et al., 2013). However, the distance between neighboring slices is usually much larger than the pixel size, which is attributed to the ability of imaging devices or time/storage/dose constraints (Neuberta et al., 2012). The direct use of such data for three-dimensional (3D) image reconstruction often results in inaccurate images due to the heterogeneous dimensions of the images, the structure of discontinuous errors, sharp points, and other errors. To obtain volumetric (3D) data with isotropic dimensions and to reconstruct the 3D structure, it is essential to conduct several interpolations between the sections (Pan et al., 2012). In the other words, as the conventional imaging devices are two-dimensional (2D), to have a 3D accurate image for better diagnosis and treatment, a set of 2D images are often taken and combined (Ebied et al., 2018). However, one of the major problems is the presence of blind or undefined dots in one or more of the images. To address this issue a 2D interpolation operation is used (Hung et al., 2019). Recently, several methods for 2D interpolation have been proposed. Some of which are discussed as follows. In (Leng et al., 2013), while expressing the problem of various categories of image interpolation methods, multiple resolution methods have been used to internalize medical images. In this method, first, a few images are taken with different resolutions and then by internalizing them, a small image is extracted but with minimal information deletion. Classical mathematical techniques use a set of basic functions to estimate the values between cuts. The nearest neighbor, B-Spline linear, and cube functions are standard types of these techniques. Such introspection approaches are commonly used in modern medical imaging (Neuberta et al., 2012). To improve the accuracy, different families of spline functions have been accepted and used as introspection cores (Pan et al., 2012). The interpolation operation itself leads to an increase in image size, but in order to store images, they must first be reduced in size so that they do not take up much space (Ebied et al., 2018). In research, interpolation methods are divided into two categories: scene-oriented and goal-oriented. Scene-based techniques are effective and easy to implement but can produce significant artifacts that relate pixels that occupy the same matrix location in continuous images to different anatomical structures. But in contrast to target-based introspection techniques, the information in the image slices is used to facilitate more accurate introspection (Hung et al., 2019). The second category is much more common and has received more attention because, for example, in an image of the brain, a mass can be considered as a target and a 3D view of the target can be obtained more accurately. In (Triwijoyo and Adil, 2021), the Bicubic interpolation algorithm is used to resize images and then by analyzing the three parameters of mean square error, mean square root of error, and maximum signal-to-sound ratio and analysis and They analyze the superiority of their work over the Bilinear method and nearest neighbor algorithms have shown. They also concluded that Bilinear and Nearest-neighbors increase the level of computational complexity (Murad et al., 2021). Presents a method based on efficient interpolated compressed sensing to increase the speed and accuracy of MRI devices. In (Iglesias et al., 2021), the deep convolutional neural network (NN) is used to internalize medical images. In the mentioned paper, the effect of variable contrasts and different orientations is considered and acceptable results are obtained. The disadvantages of the mentioned paper are time-consuming and an average square error of more than 0.01.

Today, computational intelligence has permeated most sciences (Tavoosi et al., 2011c, 2017a; Pour Asad et al., 2016, 2017). Artificial NNs have been extensively employed in the medical sciences, especially in predictive discussion (Maihami et al., 2016; Kazemi et al., 2017; Ayat, 2018; Armand et al., 2019; Tabatabaei et al., 2019). However, not much has been done in the field of interpolation of medical images using computational intelligence methods (neural network, fuzzy logic, etc.). In the following, some of the work done in this field will be reviewed. In (Chao and Kim, 2019), the fuzzy neural network of the radial base function has been used to internalize medical images. Accordingly, two suspended images are normally used to be inserted as the input of the fuzzy NN. The final output data is obtained using a learned NN. In this article, 6 entries and 3 membership functions are considered for each. Therefore, the number of fuzzy rules is 3^6^ = 243. Meanwhile, the number of neurons in the third and fourth layers is 4,374 and 729, respectively, which complicates the network, and also the execution time of the program will be very long. Naturally, such a structure will not be able to run online. A comparative plan for the development of core-based introspection methods that simultaneously improves image resolution and maintains accurate local edges is presented in Chen and Wang (2010).

Medical imaging researchers have been inspired by the advancement of deep learning methods and computational resources to combine deep learning in medical image analysis. Some recent studies have shown that accurate algorithms are successfully used to segment medical imaging and diagnose and classify diseases. A deep learning method is presented in Havaei et al. (2017), in which the network uses a circular layer instead of a fully connected layer to accelerate the segmentation process. A cascading structure is used, which compares the output of the first network with the successful network input. The network provided in Pereira et al. (2016) uses small cores to classify pixels in the image. Using small cores, without worrying about over-training (Sharifian et al., 2011), reduces the number of network parameters and helps to create deeper networks (Tavoosi et al., 2011a,b; Tavoosi et al., 2012). Increasing and normalizing its intensity has been done in the preprocessing phase to facilitate the training process. Using fuzzy theory with a genetically based learning algorithm, first the distance corresponding to the new edge is estimated based on local slope information, and then this estimated distance is used in different introspection methods instead of the main Euclidean distance. In addition, a genetic algorithm-based learning method is provided to automatically obtain important parameters of the fuzzy system. In short, for a particular pixel being processed, we replace the six input pixels. In turn, we can obtain basic data to produce an integrated interpolation image. Subsequently, several upgrade cycles were applied to train NN. Finally, we can get the desired output through the updated neural network (Tavoosi et al., 2016a,b; Tavoosi and Azami, 2019). In (Deepika et al., 2021), a fuzzy neural network has been used to compress medical images and read them quickly. The deep neural network has been used to classify and quickly read medical images (Puttagunta and Ravi, 2021). In the mentioned paper, a convolutional neural network (2D) has been used, which has a high speed, but unfortunately, it does not have good accuracy and has some errors. Type-2 fuzzy logic (T2FL) is rarely used in image interpolation, and this method is still in its infancy. For example, recently in Mohammed and Hussain (2021) a combination of Mamdani T2FL with a convolutional neural network has been used to identify images of animals. Although the mentioned article has some drawbacks such as not examining different angles, not examining blurry images, not examining the background of the same color as the animal, etc.,. However, because it is at the beginning of the path, it is generally appropriate and acceptable. In this paper, we propose a new method based on a RT2FNN. This structure consists of five layers (Tavoosi et al., 2017b). In the following, the problem will be explained first. Then we talk about the RT2FNN. In continue, the evaluation of the proposed method is presented by simulation and at the end, the conclusion is expressed.

## Statement of the Problem

**FIGURE 1:**
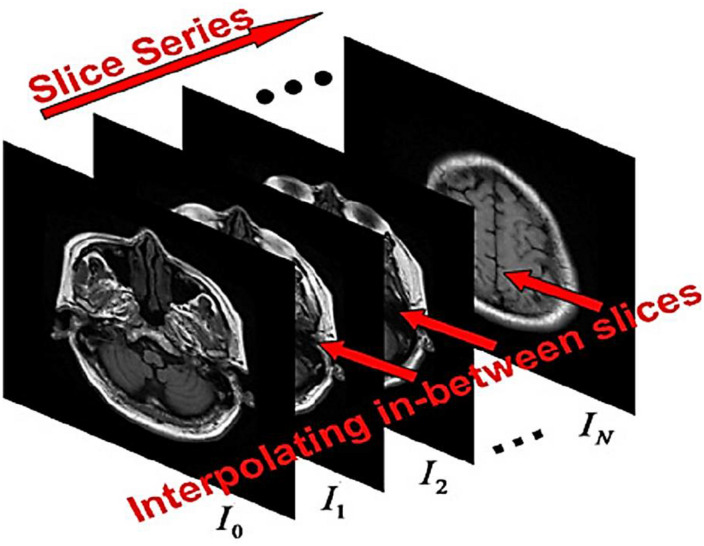
The sequence of cross-sections to create a three-dimensional image.

According to the sequence of images (called the cut) ${\left\{I_{i}\right\}}_{i=0}^{N}$ with the same size (*w* + 1) *x* (*h* + 1); where w and h are two positive integers; Figure 1). The problem of image embedding to determine the sequence of preserved properties between intersections *J*_*i*,*m*_,*i* = 0,…,*N*_1_,*m* = 1, 2,…,*M*_*i*_ so that $\overset{M_{i}}{m=1}$ {*J*_*m*,*i*_}. Continuous transfer from *I_i_* to *I*_*i+1*_. Here *M_i_* is the number of cuts inserted between *I_i_* and *I*_*i+1*_. For simplicity, assume that *M_i_* for *i* = 0.1,…, N–1. The inputs are discrete matrices. To facilitate calculations, the matrix *I* = [*I_i_j*]_(*w* + 1)×(*h* + 1)_ with size (*w* + 1)×(*h* + 1) can be used as *I*(*u*). With *u* = [*u*.*v*]^*T*^ ∈ Ω = [0,1]^2^. Here, $I\,\left(\frac{i}{w},\frac{i}{h}\right)=I_{i\,j},\left[c\,p\,s\,b\,r\,e\,a\,k\right]\,i=0,1,\ldots ,w,j=0,1,\ldots ,h$ and other values of the function are calculated by two-line (bipolar) interpolation. We define the set of points $U=\left\{u_{i\,j}={\left[\frac{i}{w},\frac{i}{h}\right]}^{T}:i=0,1,\ldots ,w,j=0,1,\ldots ,h\right\}$ as the set of pixel points. The embedding techniques are applied through the scene or object approches. For scene-based interpolation, the internal image quality values are extracted from the image quality values given in the same situations. Simple scene-based methods are easy to calculate but may results in remarkable artifacts (waste or noise). As you know, the images obtained from the detection are very linear, blurry, and obscure.

Since the information of shape and structure are not used, then the recording-based technique can be considered as an object-based technique. Note, for example, that the two images *I*_*o*_(*u*) and *I*_1_(*u*), the recording-based theme always consists of two steps; first, using a recording method to draw an image with another image, second, to create a deformation, a traditional attachment is employed.

The basic idea of image capture is to find an *X*(*u*) conversion that adapts one image to another. The similarity of the two images is considered to be the sum of the differences in square intensity (SSD), cross-correlation, and cross-sectional information. Converting *X*(*u*) can be hard or non-hard. Hard conversion is easier, and there are fewer parameters such as transmission, rotation, and scalability. However, the non-hard transfer is more flexible. *X*(*u*) transmission is usually represented by a fragmentary-linear function. To smooth out the transfer, some settings minimize the changes in the minor derivatives of the transfer. Therefore, the functional energy for the model is written as follows.

(1) $$ℰ\,\left(\text{x}\right)=𝒮\,\left(I_{0}\,\left(x\,\left(u\right)\right),I_{1}\,\left(u\right)\right)+ℛ\,\left(x\right)$$

The first semester measures the similarity between *I*_0_(*X*(*u*)) and *I*_1_(*u*), and the second semester represents the order of *X*(*u*). In the similarity statement, *I_0_* is deformed by *X*, while *I_1_* is constant. Here, the relation (1) that transforms only one image is called the one-way model.

Note that the roles of *I_0_* and *I_1_* are symmetrical in the one-way model. Suppose that the *x*_0_(*u*) map for the *I_0_* deformation is made in such a way that *I*_*o*_(*x*_0_(*u*))≈*I*_1_(*u*). Furthermore, another map *x*_1_(*u*) is constructed to meet the conditions *I*_1_(*x*_1_(*u*))≈*I*_0_(*u*). This process is called back recording. It should be noted that the hole The loop in *I_1_* cannot appear when *I_0_* is converted to *I_1_*. When *I_1_* is converted to *I_0_*, the holes become smaller. Maps *x_0_* and *x_1_* are displayed by the B_spline function and they are displayed using grids. The network map *x*(*u*)(*u* = [*u*,*t*]^*T*^ ∈ [0,1]^2^) from a vertical curve family $x\,{\left(\frac{i}{20},v\right)}_{i=0}^{20}$; and a set of horizontal curves $x\,{\left(u,\frac{i}{20}\right)}_{j=0}^{20}$ is formed. If the record is backward, and the reverse trend is facing to be forward, the equation $x_{0}=x_{1}^{-1}$ must be or estimate $x_{0}^{-1}=x_{1}$. However, we can see that forward recording is not the opposite of posterior recording, and therefore the role of *I_0_* and *I_1_* in the single-directional recording, the model is different. $J_{m\,i\,d}^{\left(01\right)}\,\left(u\right)$ is in the middle of the middle image, which is inserted based on the front recording, and the image $J_{m\,i\,d}^{\left(01\right)}\,\left(u\right)$ is in the middle of the middle image obtained by the rear recording. The derived images through the backward/forward recording are a general way to prevent saturation. However, the artificial effect (parasite) may still exist, as the images created by the two recording approaches may be quite different from each other. In the suggested method, the reshaping of both *I_0_* and *I_1_* are considered.

### Methods and Design of Algorithms

There are three steps you can take to begin the process of preparation for mediation. The suggested recording and introspection approaches are described in this section.

According to the two images *I_0_* and *I_1_* with size (*w* + 1)×(*h* + 1), we use them as continuous functions *I*_0_(*u*) and *I*_1_(*u*) and

(2) $$u={\left[u,t\right]}^{T}\in \Omega \,{\left[0,1\right]}^{2}$$

Introducing bipolar introspection. In the registration model, we are going to find two maps:

(3) $$x_{k}={\left[x_{k},y_{k}\right]}^{T}:\Omega \to \Omega ,k=0,1$$

In which case the following is established:

(i) *x*_*k*_ are *C*^2^ *maps*:

(ii)*x*_*k*_(0, *v*) = 0, *x*_*k*_(1, *v*) = 1, *y*_*k*_(*u*, 0)

       = 0 and *y*_*k*_(*u*, 1) = 1;

(iii)*for* a *given* ∈ (0 < ε < 1),

$$det\left[x_{k\,u},x_{k\,v}\right]\geq \in \text{on}\Omega .$$

where in,

(4) $$\begin{array}{c} ℰ\,\left(x_{0},x_{1}\right)=\int_{\Omega }\frac{\left[I_{0}\left(x_{0}\left(u\right)\right)-I_{1}x_{1}\left(u\right)\right)]{}^{2}}{1.0+c\,\left[I_{0}\,{\left(x_{0}\,\left(u\right)\right)}^{2}+I_{1}\,{\left(x_{1}\,\left(u\right)\right)}^{2}\right]}\,du\\ +\lambda_{1}\sum_{k=0}^{1}\int_{\Omega }\left[∥x_{k\,u}\left(u\right){∥}^{2}+∥x_{k\,v}\left(u\right)\right){∥}^{2}]du\\ +\lambda_{2}\,\sum_{k=0}^{1}\int_{\Omega }{∥x_{k\,u}\,\left(u\right)\times x_{k\,v}\,\left(u\right)∥}^{2}\,du \end{array}$$

Equation (4) must be minimized. Here *x*_*ku*_(*u*) and *x*_*kv*_(*u*) express the first partial derivatives of *x*_*k*_(*u*) relative to the variables u and v. The condition (iii) is said to be a regular condition that guarantees *x_k_* as an injection map. Set (*V*(Ω) = *X*:Ω→Ω; justifying conditions 1 to 3) Then ∀*x* ∈ *V*(Ω) is an internal map one by one.

In this paper, the maps *x*_*k*_(*u*) (*k* = 0,1) are expressed as two-variable cube elliptical functions with vector B-spline of the size defined in Ω. The first term of the energy function (4) refers to the similarity relationship used to minimize the error between the two deformed images. Regarding the inconvenience of registration appraoh, some constraints are needed to make *x*_*k*_(*u*)(*k* = 0,1) as much as possible. The second and third relations are applied to smooth the transformations *x*_*k*_(*u*)(*k* = 0,1)We set ${ℛ}_{1}\sum_{k=0}^{1}\int_{\Omega }\left[∥x_{k\,u}\left(u\right){∥}^{2}+∥x_{k\,v}\left(u\right)\right){∥}^{2}]du$ two relationships Call the first time and go to ${ℛ}_{2}\,\left(x_{0},x_{1}\right)\,\sum_{k=0}^{1}\int_{\Omega }\left({∥x_{k\,u}\times x_{k\,v}∥}^{2}\right)\,du$ Let’s say that the parameters λ_1_ and λ_2_ are two specific coefficients of regulatory expressions. The following is an interpretation and analysis of the model:

### The Relationship of Similarity

The two maps *x*_0_(*u*) and *x*_1_(*u*) are designed to reshape *I_0_* and *I_1_* in such a way that the reshaped images of *I*_*k*_(*x*_*k*_(*u*))(*k* = 0,1) are similar. Compared to one-dimensional recording, the use of two maps in the same period overcomes the saturation problem *I_0_* and *I_1_*. Also, images *I*_0_(*x*_*u*_(*u*)) and *I*_1_(*x*_1_(*u*)) match using only one map, because the free parameters has doubled.

To measure the similarity between the two images *I_0_* and *I_1_*, a simple metric and a cheap computation, the SSD is ∫_Ω_(*I*_0_(*u*)−*I*_1_(*u*))^2^d*u*. However, the endurance of non-compliance for the area of difference between the intensity of the squares of the low-intensity area is greater than that of the more intense area. In practical applications, low-intensity features are as important as high-intensity features. In practical applications, low-intensity features are as important as high-intensity features. Therefore, a modified criterion ∫_Ω_*g*(*u*)(*I*_0_(*u*)−*I*_1_*u*))^2^*du* (SSD) is applied to our model. The term *g*(*u*) = 1/[1.0 + *c*(*I*_0_(*u*)^2^ + *I*_1_(*u*)^2^] is denoted by the denominator of at least 1 and c as a constant positive number.

### The Relationship of the First Order Settings

For the desired mapping *x*(*u*), $\int_{0}^{1}∥x_{u}\,\left(u,v_{0}\right)∥\,du$ is the length of the arc curve *C*(*u*):=*x*(*u*,*v*_0_). Also $\int_{0}^{1}∥x_{v}\,\left(u_{0},v\right)∥\,dv$ and the length of the arc curve is *C*(*v*):=*x*(*u*_*v*_,*v*). Therefore, the setting of the first-order expression ∫_Ω_∥*x*_*u*_(*u*)^2^∥ + ∥*x*_*v*_(u)∥^2^*du* intends to map the conversion of *x* according to the variables *u* and *v*. The phrase “regulator” denotes a convex function based on *x*, so we can get at least the predicate if and only if *x* is the same mapping.

### Phrase Regional Settings

In parametric form *x*:Ω⟶Ω, the surface element is written by ∥*x*_*u*_×*x*_*v*_∥*du*. For Ω = [0,1]^2^, the area *xεV*(Ω) is equal to: |Ω| = ∫_Ω_∥*x*_*u*_×*x*_*v*_∥d*u* = 1. Using the inequality Kushi_Shuartz:

$1=\int_{\Omega }∥x_{u}\times x_{v}∥du\leq \left(\int_{\Omega }∥x_{u}\times x_{v}{∥}^{2}du^{\frac{1}{2}}\right).|{|}^{\frac{1}{2}}$, The equation is established if and only if ∥*x*_*u*_×*x*_*v*_∥≡1. Thus, the relational constraints of the ∫_Ω_∥*x*_*u*_×*x*_*v*_∥^2^d*u* constraints the constraints so that the area element remains constant. Since ∥*x*_*u*_×*x*_*v*_∥^2^ = ∥*x*_*u*_∥^2^∥*x*_*v*_∥^2^− < *x*_*u*_,*x*_*v*_ >^2^, the relationship of the regional settings can be ∫_Ω_(∥*x*_*u*_∥^2^∥*x*_*v*_∥^2^- < *x*_*u*_,*x*_*v*_ >^2^)*du* also wrote.

### Select the Parameters λ_1_ and λ_2_ in the First-Order Settings

**FIGURE 2:**
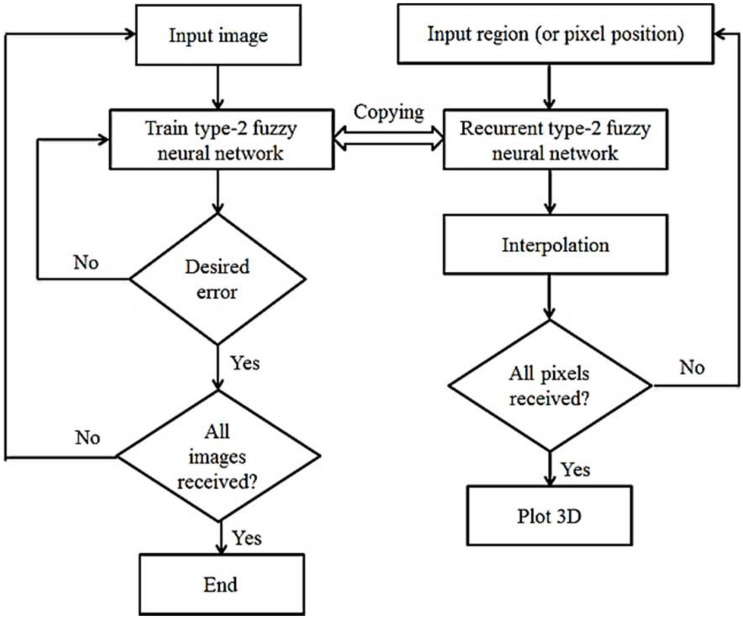
Flowchart of the proposed method.

The choice of parameters λ_1_ and λ_2_ depends on the deformation between the two specific images. The first-order setting term ℛ_1_ monitors the flexibility of *x*_*k*_(*k* = 0,1) conversions. Therefore, we have to consider λ_1_ as large if the image has high strength, and vice versa, if we consider λ_1_ to be small, there are many differences between *I_0_* and *I_1_*. Relationship settings ||*x*_*ku*_×*x*_*kv*_|| = |det⁡[*x*_*ku*_,*x*_*kv*_]| for *k* = 0,1 Limits area elements (triple conditions). Therefore, with a small λ_2_, the setting process may have to be stopped because the three conditions are not satisfactory. However, these images cannot match well with a large λ_2_, because the elastic deformation is stopped by the ℛ_2_ regulator. The registration approach is not very sensitive to regulatory parameters. In our experiments, the parameters λ_1_ and λ_2_ are experimentally selected based on the given images. Figure 2 shows the general flowchart of the work. In this figure, the left part is related to the learning phase of the recurrent type-2 neural network and the right part is related to the interpolation phase using it.

The procedure according to Figure 2 is that first the existing images are entered into the system one by one, processed and the RT2FNN is trained. The condition for completing the training is to achieve the minimum desired error. Then, in the second phase, the blind spots or pixels that are not available are trained, generated and the interpolation operation is completed by the type 2 recursive neural network, and finally, if all the pixels are identified, a 3D image printing command is issued.

## Recurrent Type-2 Fuzzy Neural Network Model

**FIGURE 3:**
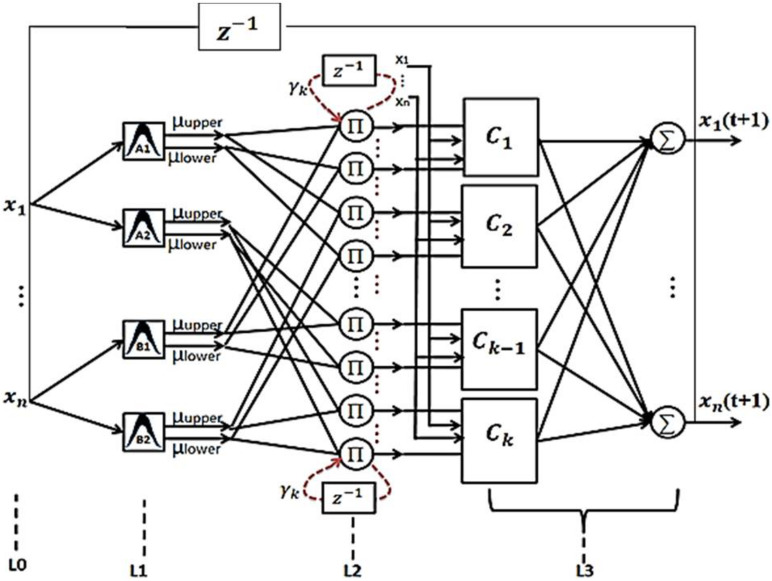
The proposed recurrent type-2 fuzzy neural network structure.

The structure of the proposed RT2FNN is shown in Figure 3.

Details of how the proposed network works and how to train it are described in Tavoosi et al. (2017b). Based on a specific pixel of the recorded image, six pixels (3–3) are selected from the two conditional images. Each pixel is represented by 3 fuzzy subsets. Then, based on the condition formulation layer, a general fuzzy rule is written by the combination of 6 fuzzy subsets. We determine the rule. By random assignment, (6 × 729) 4,374 some conditions define all the relevant rules. The number of neurons in the formulation layer is 4374 and number of rules is 729. Finally, the output pixel is calculated. Sequentially, we can obtain all the output data needed to embed an image by processing the total recorded image.

## Interpolation Using Recurrent Type-2 Fuzzy Neural Network

**FIGURE 4:**
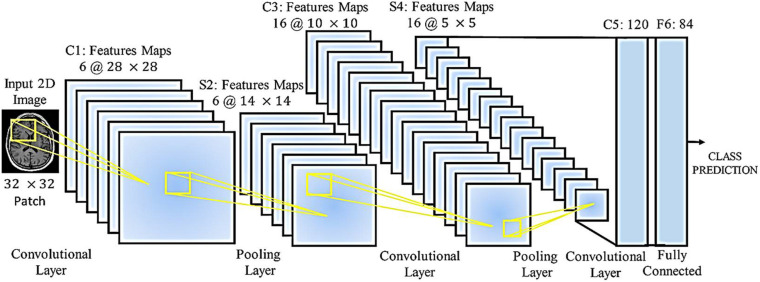
Type-2 Fuzzy neural network operation for categorizing and processing brain images.

A wide range of medical imaging techniques are used to predict and diagnose clinical problems, but in most cases, the images obtained are similar, with deep learning where the network structure allows this, gives us solutions. Once specialized knowledge is available in this field, then the manipulated features operate, and in general it can be said that this creates difficult and complex assumptions, and these assumptions may be for some. Do not use medical imaging. So despite the hand-made features, it’s hard to tell the difference between healthy and unhealthy images in some cases. A classifier such as a support device (SVM) does not provide a final and comprehensive solution. Features derived from methods such as the criterion for converting immutable properties (SIFT) are independent of the task or task assigned. Classifiers such as vector support have been applied to this model, and no mechanism has been improved to lose local features, which in the process of extracting features and classifying those that are separated from each other, does not exist. On the other hand, a RT2FNN learns these properties through basic data. These features are guided data and are learned to end the learning mechanism. The ability of the fuzzy neural network to regenerate is that the error signal obtained in lost functions is extracted and reused to improve properties (fuzzy-type recursive fuzzy-type fuzzy network filters in layers primary are taught). Therefore, RT2FNNs become a better model. Another advantage is that in the early layers of a RT2FNN, the edges surround the spots and in the local structure, while the nerve cells in the upper layers focus more on the part. Different people have human organs, some of which can completely consider the human organs in the final layers. Figure 4 shows the processing of brain images using a neural network.

Figure 4 shows the medical images for the classification of medical images by accepting a 32 × 32 N class fragment from the original 2D image. The network has loops, maximum volume, and fully connected layers. Each annular layer produces a linear design of different sizes, and the volume of the layers reduces the size of the linear designs to be transferred to the lower layers. Fully connected layers produce the prediction of the intended class at the output. Several parameters require a network, which depends on the number of layers, the number of nerve cells in each layer, and the relationship between these nerve cells.

The training phase of the network ensures that the maximum possible efficiency is learned in which the best performance is possible to solve the desired problem.

## Simulation

**FIGURE 5:**
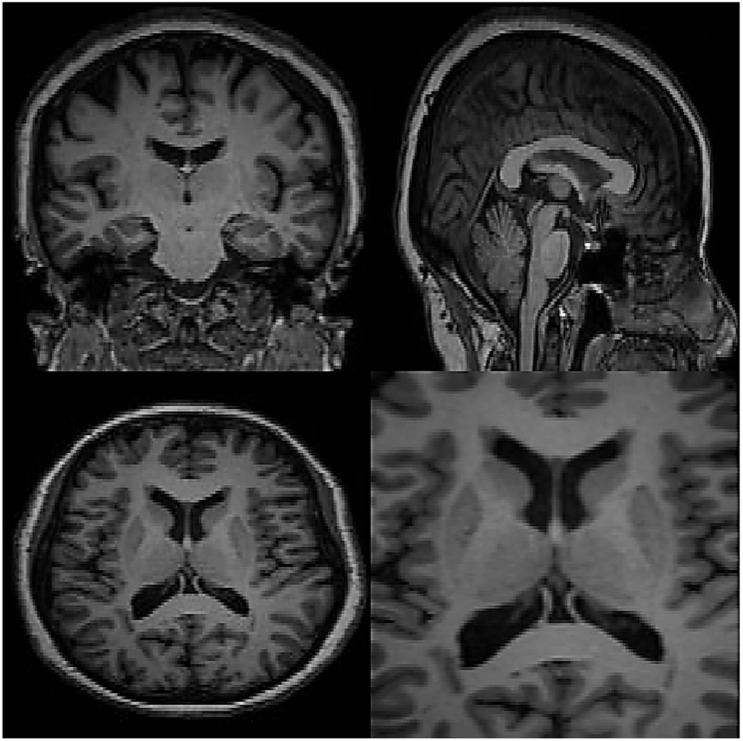
The results of recurrent type-2 fuzzy neural network for two-dimensional interpolation.

In this section, the data for brain reconstruction images are first extracted from the Allen Brain Atlas Database. The size of the images can be from 256 × 256 pixels to more than 4,000 × 4,000 pixels. Our algorithm can work with any size. The larger the size, the longer the processing time, but the higher the accuracy. In this article, we have used 100 images of 768 × 578 (442 kB) to create 3D images. The neural network has 5 intermediate layers, each layer having 100 neurons. Then, these 2D images were used to teach the RT2FNN and normal one. Figure 3 shows the results of RT2FNNs for 2D interpolation. Figure 5 shows the results of a typical type-2 fuzzy neural network for 2D interpolation. Note that a normal (typical) network does not have feedback, i.e., it does not use past moment data. In fact, one of the purposes of this article is to show the importance and impact of the presence or absence of feedback in the structure of the neural network.

The specifications of the computer users are as follows: Windows 10 Home 64; 11th Gen Intel^®^ Core^TM^ i7 processor; Intel^®^ Iris^®^ Xe Graphics; 8 GB memory; 256 GB Intel^®^ SSD Storage; 16 GB Intel^®^. The simulation was performed in MATLAB software version 2019a.

**FIGURE 6:**
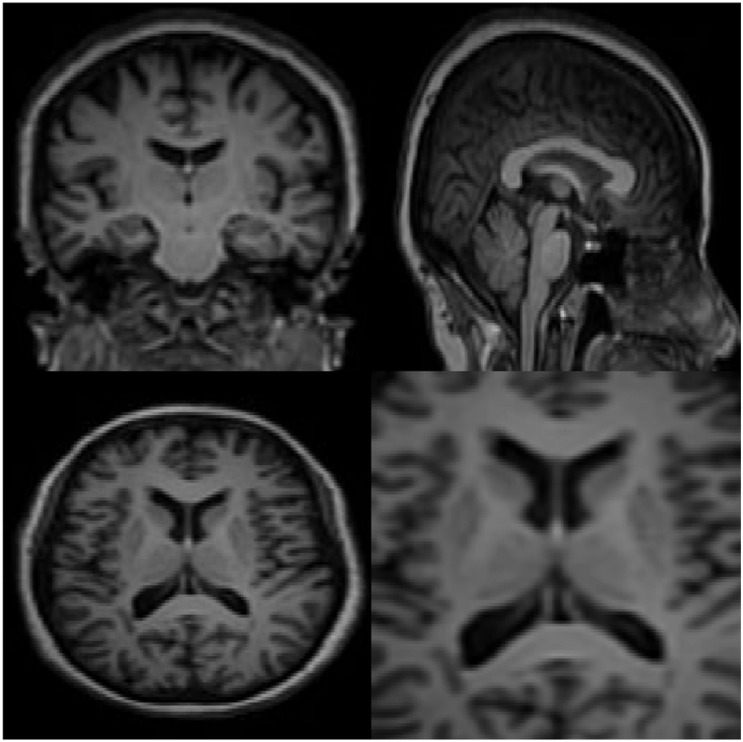
The results of a typical type-2 fuzzy neural network for two-dimensional interpolation.

**FIGURE 7:**
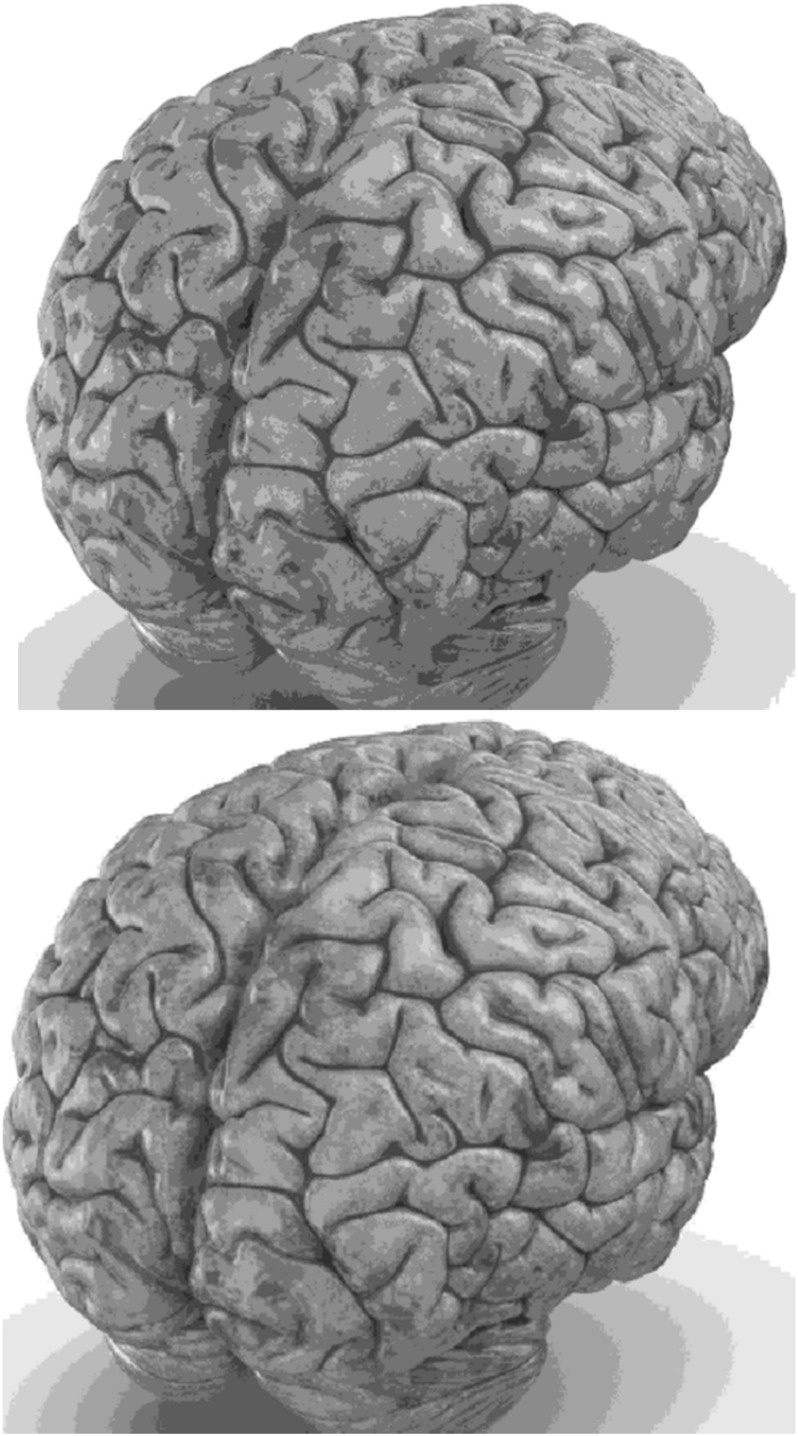
3D image of the rear view angle.

**FIGURE 8:**
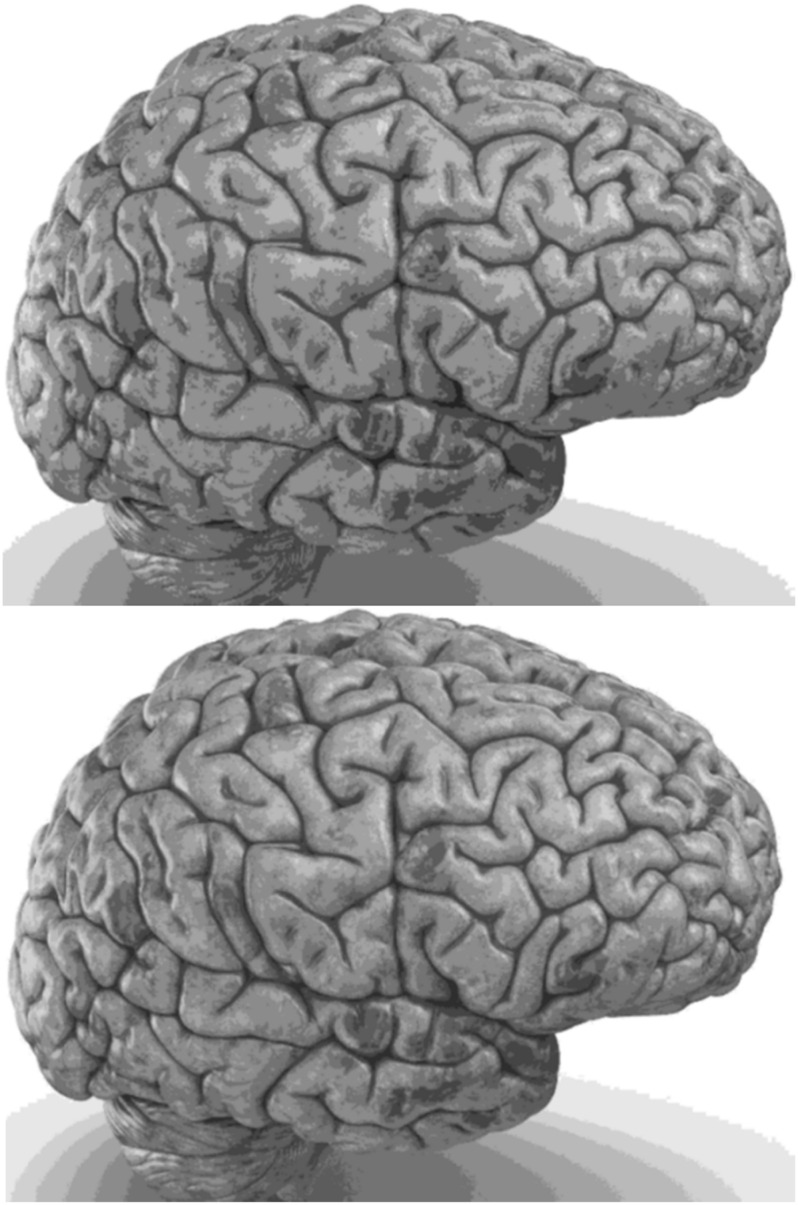
3D image from the right viewing angle.

**FIGURE 9:**
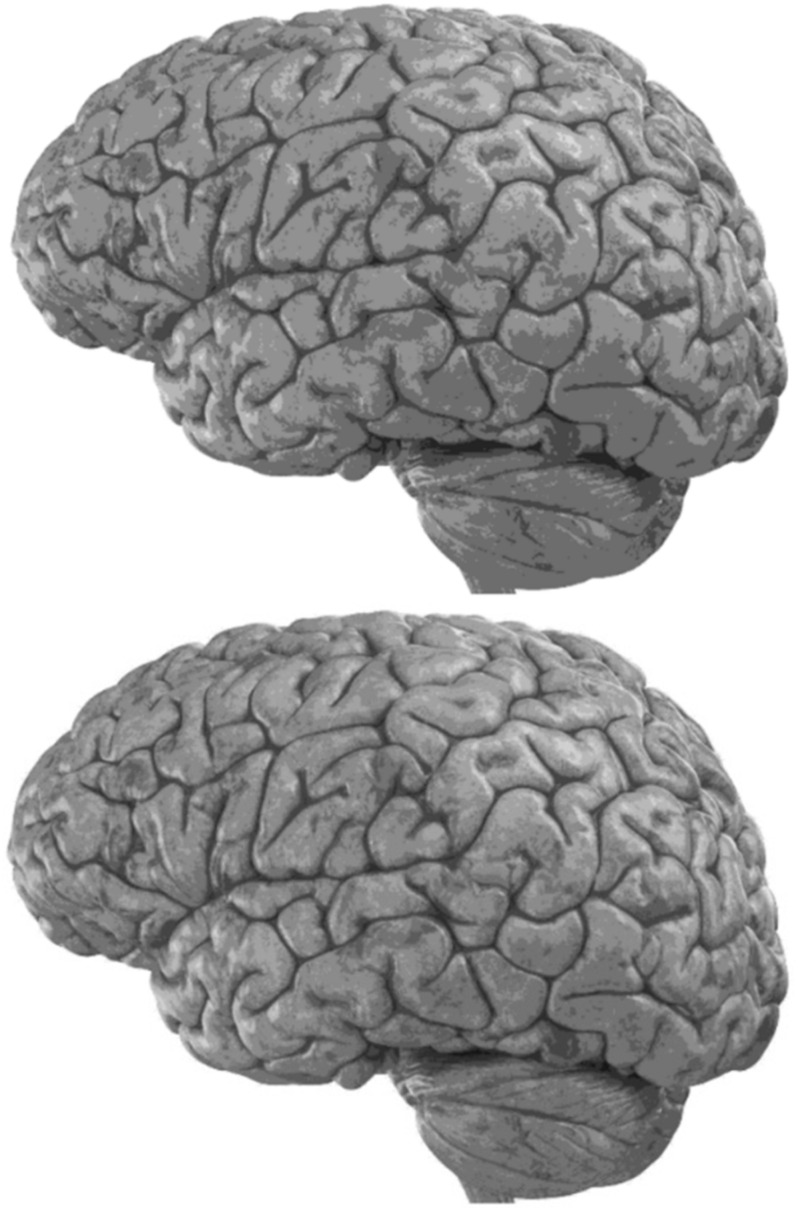
3D image from the left viewing angle.

**FIGURE 10:**
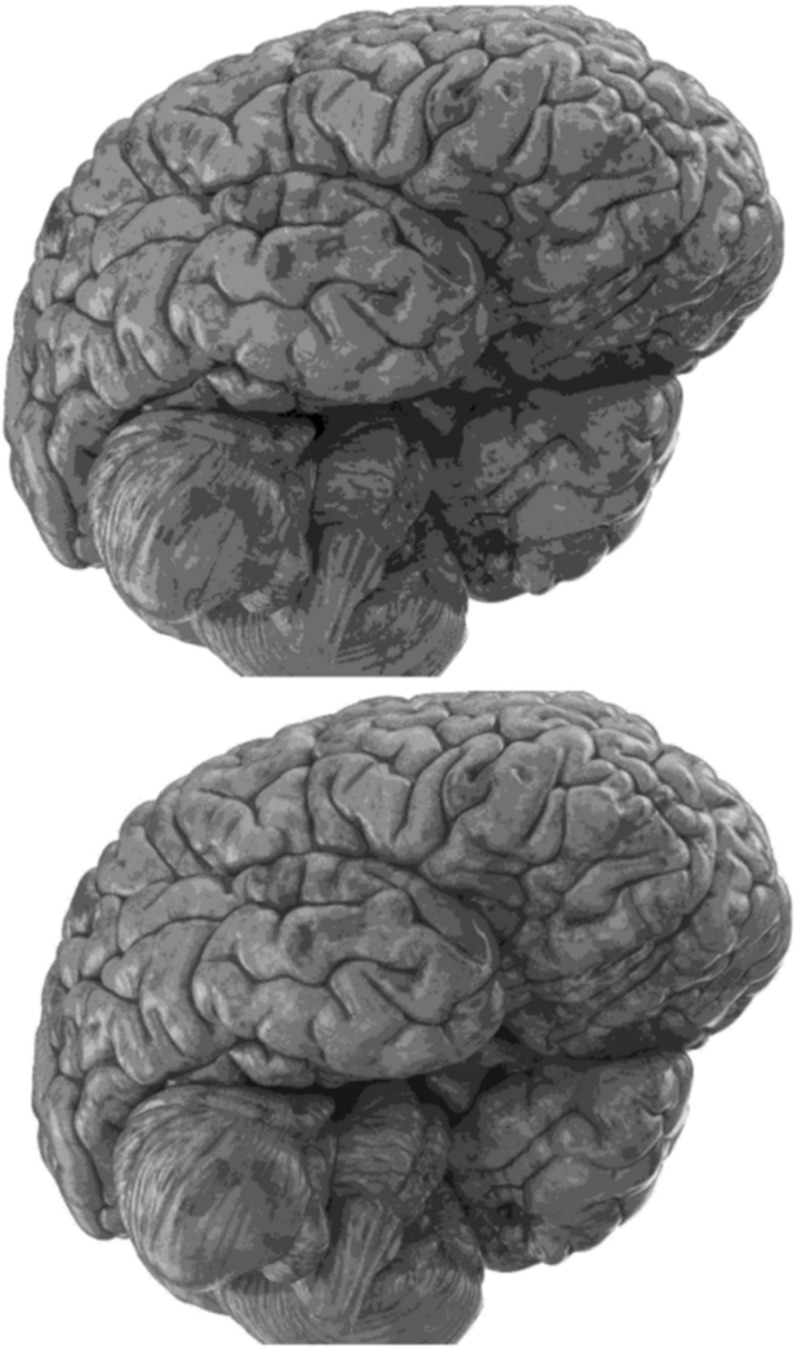
3D image from the front viewing angle.

Carefully in Figures 5, 6, it can be seen that the recurrent network has performed better, especially in detail. In the following, the ability of the recurrent network and the normal network to reconstruct the 3D image of the brain is examined. The 3D image of the brain is depicted from four angles. Figure 7, which is shown from a rearview angle, the above figure is the results from the recurrent network and the bottom figure is the results from the normal network. Also shown in Figures 8–10 are 3D images of the brain from the right, left, and front viewing angles, respectively. In all images, the above figure is for the recurrent network and the bottom figure is for the typical network.

## Discussion

The main purpose of this paper is to show the importance of feedback and the use of past moment data in the structure of type-2 fuzzy neural networks. Of course, for the first time, these networks have been used for interpolation in medical images, and this is another innovation of this paper. Naturally, the more accurate the 3D image, the easier it is for doctors and relevant specialists to work with the least error. Looking at Figures 5–10, it can be seen that the accuracy of recurrent networks is objectively higher than normal networks, and the reason for this is that recurrent networks use the information of neighboring points in the images. In Figures 8–10, as can be seen in blind spots or deep spots (indent), the difference between a recurrent and a normal network becomes more pronounced. This is especially true in Figure 10, as this image has many dents and blind spots. The reason for the superiority of the RT2FNN over conventional is the simultaneous use of output and input data to the network, while in the conventional network only input information is used. In other words, the return network has a dynamic structure, but the normal network operates statically. The network specifications used are shown in Table 1.

**TABLE 1:** Specifications of networks used.

	Number of fuzzy rules	Average squares error	Training time
Recurrent network	16	0.016	570 s
Typical network	22	0.025	510 s
Method of Neuberta et al. (2012)	–	0.15	4 s

As can be seen in Table 1, the number of fuzzy rules of the recurrent network is less than the typical network. The average error squares in the return network are far less than the typical type. But the training time of recurrent networks is longer than the training time of the typical network, which is due to the existence of feedback and its calculations. For further comparison, we also used methods without the use of computational intelligence (fuzzy logic, neural network, etc.). For example, you can see the results of the method presented in Neuberta et al. (2012) in the table. The average squares error is much higher than the intelligence-based methods, but the processing time is surprisingly short. In general, time can be sacrificed for accuracy, because a high-quality 3D image is more needed by the medical community than the time it takes to create and produce this image.

## Conclusion

In this study, the problem of interpolation in medical images using RT2FNNs was addressed. Image interpolation is used for two main purposes. Firstly, to increase the quality of images adding the number of pixels is studied. Secondly, 3D images are produced. The recurrent type-2 fuzzy neural networks model outperforms the type-2 fuzzy neural networks model. The average squares error for the recurrent network and the typical network reported 0.016 and 0.025, respectively. On the other hand, the number of fuzzy rules for the recurrent network and the typical network reported 16 and 22, respectively. The recurrent type-2 fuzzy neural network has internal feedback, and as it uses output information it can therefore provide more accurate interpolation with less error. It is worth mentioning that the training time of the images for the recurrent type-2 fuzzy neural networks model is longer. However, in the medical sciences, model accuracy is more important. It is expected that the methodology represented in this research would be extended further in 3D printers, tumor surgeries, and so on. For future studies using type-3 fuzzy logic and color images will be considered.

## Data Availability Statement

Publicly available datasets were analyzed in this study. This data can be found here: https://openfmri.org/dataset/.

## Ethics Statement

The Ethics Committee of Ilam University approved the study.

## Author Contributions

JT: writing-original draft preparation, software, validation, visualization, investigation, and supervision. CZ and AM: writing-original draft editing, visualization, investigation, and supervision. AM: writing-original draft editing, software, validation, visualization, and investigation. SM: writing-original draft editing, validation, software, and validation. All authors contributed to the article and approved the submitted version.

## Conflict of Interest

The authors declare that the research was conducted in the absence of any commercial or financial relationships that could be construed as a potential conflict of interest.

## Publisher’s Note

All claims expressed in this article are solely those of the authors and do not necessarily represent those of their affiliated organizations, or those of the publisher, the editors and the reviewers. Any product that may be evaluated in this article, or claim that may be made by its manufacturer, is not guaranteed or endorsed by the publisher.

## References

[B1] Armand R. Rigi G. Bahrami T. (2019). Fuzzy hybrid least-squares regression approach to estimating the amount of extra cellular recombinant protein A from *Escherichia coli* BL21. *J. Ilam Univ. Med. Sci.* 27 1–13. 10.29252/sjimu.27.3.1 30186992

[B2] Ayat S. (2018). Increasing the speed and precision of prediction of the results of angiography by using combination of adaptive neuro-fuzzy inference system and particle swarm optimization algorithm based on data from Kowsar Hospital of Shiraz. *J. Ilam Univ. Med. Sci.* 26 142–154. 10.29252/sjimu.26.4.142 30186992

[B3] Chao Z. Kim H. J. (2019). Slice interpolation of medical images using enhanced fuzzy radial basis function neural networks. *Comput. Biol. Med.* 10 66–78. 10.1016/j.compbiomed.2019.05.013 31129416

[B4] Chen H. C. Wang W. J. (2010). Locally edge-adapted distance for image interpolation based on genetic fuzzy system. *Expert Syst. Appl.* 37 288–297. 10.1016/j.eswa.2009.05.069

[B5] Deepika J. Rajan C. Senthil T. (2021). Security and privacy of cloud- and IoT-based medical image diagnosis using fuzzy convolutional neural network. *Comput. Intell. Neurosci.* 2021 1–17. 10.1155/2021/6615411 33790958 PMC7997756

[B6] Ebied M. Elmisery F. A. Zekry A. (2018). “Utilization of decimation interpolation strategy for medical image communication and storage,” in *Proceedings of the 2018 8th International Conference on Computer Science and Information Technology (CSIT)*, Amman, 22–25.

[B7] Havaei M. Davy A. Warde-Farley D. Biard A. Courville A. Bengio Y. (2017). Brain tumor segmentation with deep neural networks. *Med. Image Anal.* 35 18–31. 27310171 10.1016/j.media.2016.05.004

[B8] Hung K. W. Wang K. Jiang J. (2019). Image interpolation using convolutional neural networks with deep recursive residual learning. *J. Multimed. Tools Appl.* 78 22813–22831. 10.1007/s11042-019-7633-1

[B9] Iglesias J. E. Billot B. Balbastre Y. Tabari A. Conklin J. González R. G. (2021). Joint super-resolution and synthesis of 1 mm isotropic MP-RAGE volumes from clinical MRI exams with scans of different orientation, resolution and contrast. *Neuroimage* 237:118206. 10.1016/j.neuroimage.2021.118206 34048902 PMC8354427

[B10] Kazemi M. Mehdizadeh H. Shiri A. (2017). Heart disease forecast using neural network data mining technique. *J. Ilam Univ. Med. Sci.* 25 20–32. 10.29252/sjimu.25.1.20 30186992

[B11] Leng J. Xu G. Zhan Y. (2013). Medical image interpolation based on multi-resolution registration. *Comput. Math. Appl.* 66 1–18. 10.1016/j.camwa.2013.04.026

[B12] Maihami V. Khormehr A. Rahimi E. (2016). Designing an expert system for prediction of heart attack using fuzzy systems. *Sci. J. Kurdistan Univ. Med. Sci.* 21 118–131.

[B13] Mohammed H. R. Hussain Z. M. (2021). Hybrid mamdani fuzzy rules and convolutional neural networks for analysis and identification of animal images. *Computation* 9:35. 10.3390/computation9030035

[B14] Murad M. Jalil A. Bilal M. Ikram S. Ali A. Khan B. (2021). Radial undersampling-based interpolation scheme for multislice CSMRI reconstruction techniques. *Biomed Res. Int.* 2021:6638588. 10.1155/2021/6638588 PMC8057880 33954189

[B15] Neuberta A. Salvado O. Acosta O. Bourgeat P. Fripp J. (2012). Constrained reverse diffusion for thick slice interpolation of 3D volumetric MRI images. *Comput. Med. Imaging Graph.* 36 130–138. 10.1016/j.compmedimag.2011.08.004 21920702

[B16] Pan M. S. Yang X. L. Tang J. T. (2012). Research on interpolation methods in medical image processing. *J. Med. Syst.* 36 777–807. 10.1007/s10916-010-9544-6 20703653

[B17] Pereira S. Pinto A. Alves V. Silva C. A. (2016). Brain tumor segmentation using convolutional neural networks in mri images. *IEEE Trans. Med. Imaging* 35 1240–1251. 10.1109/tmi.2016.2538465 26960222

[B18] Pour Asad Y. Shamsi A. Ivani H. Tavoosi J. (2016). Adaptive intelligent inverse control of nonlinear systems with regard to sensor noise and parameter uncertainty (magnetic ball levitation system case study). *Int. J. Smart Sens. Intell. Syst.* 9 148–169. 10.21307/ijssis-2017-864

[B19] Pour Asad Y. Shamsi A. Tavoosi J. (2017). Backstepping-based recurrent type-2 fuzzy sliding mode control for MIMO systems (MEMS triaxial gyroscope case study). *Int. J. Uncertain. Fuzziness Knowl. Based Syst.* 25 213–233.

[B20] Puttagunta M. Ravi S. (2021). Medical image analysis based on deep learning approach. *Multimed. Tools Appl.* 80 24365–24398. 10.1007/s11042-021-10707-4 PMC8023554 33841033

[B21] Sharifian M. B. B. Mirlo A. Tavoosi J. Sabahi M. (2011). “Self-Adaptive RBF Neural Network PID Controller in Linear Elevator,” in *Proceedings of the International Conference on Electrical Machines and Systems*, Beijing.

[B22] Tabatabaei S. M. R. Saadatjoo F. Mirzaei M. (2019). The prediction model for cardiovascular disease using Yazd’s health study data (YaHS). *J. Shahid Sadoughi Univ. Med. Sci.* 27 1346–1360.

[B23] Tavoosi J. Alaei M. Jahani B. (2011a). “Temperature Control of Water Bath by Using Neuro-Fuzzy Controller,” in *Proceedings of the 5th Symposium on Advance in Science and Technology*, Mashhad.

[B24] Tavoosi J. Alaei M. Jahani B. Daneshwar M. A. (2011b). A novel intelligent control system design for water bath temperature control. *Aust. J. Basic Appl. Sci.* 5 1879–1885.

[B25] Tavoosi J. Azami R. (2019). A new method for controlling the speed of a surface permanent magnet synchronous motor using fuzzy comparative controller with hybrid learning. *J. Comput. Intell. Electr. Eng.* 10 57–68.

[B26] Tavoosi J. Badamchizadeh M. A. Ghaemi S. (2011c). Adaptive inverse control of nonlinear dynamical system using type-2 fuzzy neural networks. *J. Control* 5 52–60.

[B27] Tavoosi J. Shamsi Jokandan A. Daneshwar M. A. (2012). A new method for position control of a 2-DOF robot arm using neuro-fuzzy controller. *Indian J. Sci. Technol.* 5 2253–2257.

[B28] Tavoosi J. Suratgar A. A. Menhaj M. B. (2016a). Nonlinear system identification based on a self-organizing type-2 fuzzy RBFN. *Eng. Appl. Artif. Intell.* 54 26–38. 10.1016/j.engappai.2016.04.006

[B29] Tavoosi J. Suratgar A. A. Menhaj M. B. (2016b). Stable ANFIS2 for nonlinear system identification. *Neurocomputing* 182 235–246. 10.1016/j.neucom.2015.12.030

[B30] Tavoosi J. Suratgar A. A. Menhaj M. B. (2017a). Stability analysis of recurrent type-2 TSK fuzzy systems with nonlinear consequent part. *Neural Comput. Appl.* 28 47–56. 10.1007/s00521-015-2036-3

[B31] Tavoosi J. Suratgar A. A. Menhaj M. B. (2017b). Stability analysis of a class of MIMO recurrent type-2 fuzzy systems. *Int. J. Fuzzy Syst.* 19 895–908. 10.1007/s40815-016-0188-7

[B32] Triwijoyo B. K. Adil A. (2021). Analysis of medical image resizing using bicubic interpolation algorithm. *J. Ilmu Komput.* 14 20–29.

